# Dramatic Response of Leptomeningeal Carcinomatosis to Nivolumab in PD-L1 Highly Expressive Non-small Cell Lung Cancer: A Case Report

**DOI:** 10.3389/fonc.2019.00819

**Published:** 2019-09-06

**Authors:** David Arias Ron, Carmen M. Labandeira, María Carmen Areses Manrique, Paula Sampedro Domarco, Ihab Abdulkader, Jesús García-Mata, Christian Rolfo, Diego González-Rivas, Jose Luis Fírvida

**Affiliations:** ^1^Medical Oncology Department, University Hospital Complex of Ourense, Ourense, Spain; ^2^Neurology Department, University Hospital Complex of Vigo, Hospital Alvaro Cunqueiro, Pontevedra, Spain; ^3^Anatomopathology Department, University Hospital Complex of Santiago de Compostela, Santiago de Compostela, Spain; ^4^Thoracic Medical Oncology, Early Clinical Trials, University of Maryland Marlene and Stewart Greenebaum Comprehensive Cancer Center, Baltimore, MD, United States; ^5^Thoracic Surgery Department, Hospital San Rafael, Coruña, Spain; ^6^Department of Thoracic Surgery, Shanghai Pulmonary Hospital, Shanghai, China

**Keywords:** squamous lung cancer, nivolumab, leptomeningeal carcinomatosis, PD-L1, complete response

## Abstract

In a patient who had been diagnosed of located squamous cell lung carcinoma, pneumonectomy, and adjuvant chemotherapy were performed. Brain recurrence and subsequent lung metastatic disease were uncontrolled by neurosurgery, holocranial radiotherapy, and first-line chemotherapy. In August 2015, appearance of leptomeningeal carcinomatosis triggered severe clinical deterioration and threatened the patient's life. Anti-PD1 immune checkpoint inhibitor *Nivolumab* was initiated in an attempt to stop tumor growth, achieving a spectacular brain and pulmonary complete response and clinical improvement, without serious adverse effects. High expression PD-L1 level (100%) was found in the pathological tissue sample. Nivolumab was maintained for more than 2 years and stopped in December 2017 after 28 months of treatment, with no disease evidence. More than 3 years after its onset, the patient maintains an outstanding PS with complete tumor response and no evidence of disease in last surveillance CT scan and brain MRI.

## Background

Non-small cell lung cancer (NSCLC) encompasses 85% of total lung malignancies, which remains to be the leader malignancy worldwide accounting more than 1.5 million of deaths every year (1). During a long time, cisplatin-doublets were the most used treatment in metastatic disease, with poor median survival of 7.9 months (2), and our options after progression were mournfully limited, as docetaxel monotherapy (3). However, in the last decade, our knowledge about oncogenic driver mutations and immune checkpoint inhibitors suffered an outbursting growth which has changed radically the therapeutic landscape of our patients. EGFR mutations and ALK gene rearrangements, first, but specially immunotherapy, later, has explored new targetable pathways in our fight against lung cancer (4).

Immune checkpoint blockades have demonstrated enhanced tumor regression by reversing tumor-induced immunosuppression, leading immune cells to recognize and eliminate cancer cells. Nivolumab, a fully human IgG4 monoclonal antibody, can behave like an immunomodulator by blocking ligand activation of programmed cell death 1 (PD-1) receptor on T cells, promoting antitumor activity. It was the first immune checkpoint inhibitor approved for second-line treatment of NSCLC, showing encouraging antitumor activity among previously treated patients with advanced-stage lung tumors in two phase III trials including squamous (5) and non-squamous (6) histology, compared against docetaxel; PD-L1 expression was evaluated as a predictive biomarker in both trials. Surprisingly, predictive correlation toward enhanced efficacy was clearly observed in case of PD-L1 positive non-squamous tumors, but not in case of squamous histology, achieving similar benefit regardless of PD-L1 expression. Notwithstanding, a trend toward prolonged survival and diminish of adverse events (AE) support the use of nivolumab instead of docetaxel as second-line treatment. Moreover, results obtained with different immunomodulatory drugs as pembrolizumab in first (7) and second-line treatment (8), and more recently atezolizumab (9), could suggest that PD-L1 remains the best lung immune biomarker at the moment.

Recent outcomes of 2 years follow-up in *CheckMate 017* (10) have confirmed a benefit in OS (23 vs. 8%) with nivolumab vs. docetaxel, and also a 37% of durable responses, after at least 24 months (regardless of PD-L1 expression), maintaining a lower rate of adverse events, highlighting 10 vs. 55% in case of grade 3–4 AE. Here we present the case of a patient with metastatic squamous lung cancer who achieved a complete response of pulmonary and leptomeningeal disease after more than 36 months of second-line therapy with nivolumab; significantly, PD-L1 expression showed an outstanding 100% expression level in the first tissue sample. We aim to discuss the medical steps in an attempt to continue evolving our understanding of immunotherapy role in this subset.

## Case Presentation

In January 2014, a 48-years-old white, heavily smoker man without medical history of interest presented to our hospital due to subacute fatigue and moderate haemoptysis. A chest x-ray showed a left lung lesion in the upper lobe, confirmed by subsequent positron emission tomography (PET) scan, enlarging at least one hilar left node. The anatomopathological diagnosis after bronchoscopy was squamous carcinoma of the lung, showing promising surgical options according to operability and resectability criteria. Left pneumonectomy with systematic lymphadenectomy by Uniportal Video-Assisted Thoracic Surgery (VATS) was performed in March 2014, obtaining a pT2b pN1 M0 TNM stage. Adjuvant chemotherapy was proposed, and the patient completed four cycles of Cisplatin-Vinorelbine with no disease evidence in the first CT control scan.

Eight months after chemotherapy, our patient developed vertigo, dizziness and tensional headache; MRI brain demonstrated a left single cerebellar mass of 50 × 25 mm with no other systemic disease in CT scan. Complete resection with no microscopic residual tumor was carried out by the Neurosurgery team in February 2015; complete management, individualizing this patient as a rapid relapse after adjuvant chemotherapy, included whole brain radiotherapy with dose fractionation regimen of 30 Gy delivered in 10 fractions over the course of 2 weeks, and subsequent systemic treatment with monotherapy gemcitabine, which started in March 2015.

**Figure 1:**
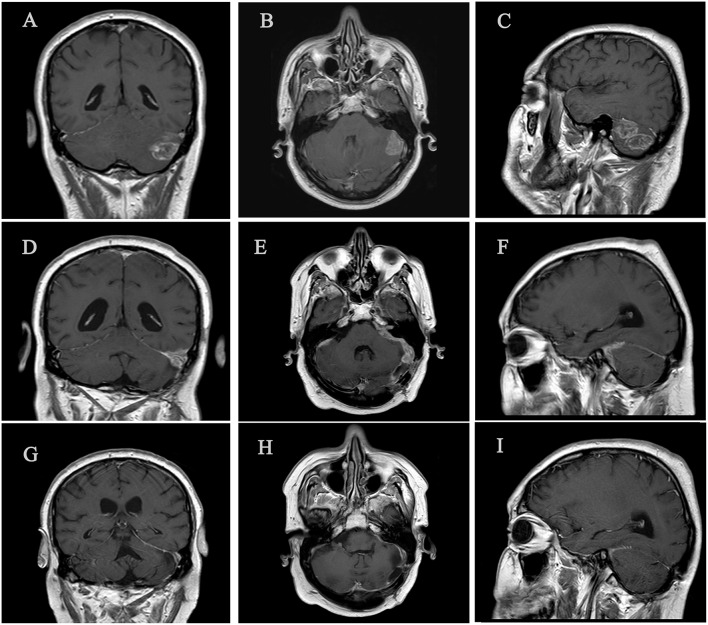
Three significant time points of brain disease. **(A–C)** Left cerebellar metastasis prior to brain surgery (January 2015). **(D-F)** Leptomeningeal metastasic disease, predominantly left cerebral hemisphere (September 2015). **(G–I)** Complete brain response with no evidence of leptomeningeal involvement after 3 months of nivolumab treatment (December 2015).

**Figure 2:**
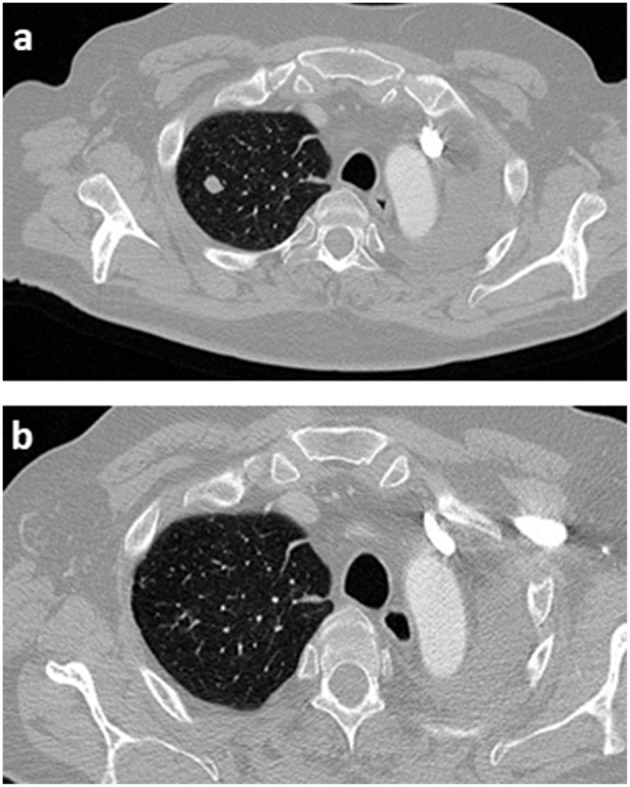
Follow-up of right lung disease before and after immunotherapy. **(a)** Pulmonary right nodule, suggesting contralateral lung disease. **(b)** Complete lung response during nivolumab treatment.

Poor news were found in July 2015. CT control scan showed at least seven pulmonary nodules affecting the upper and lower right lobes (see Figure 1); in the multidisciplinary committee, surgery was rejected because of multilobar contralateral disease. According with results of Checkmate 017 trial published on July 9, 2015, we decided to request the anti-PD1 Nivolumab in terms of compassionate use. Hopelessly, during the 3 weeks until approval, the patient presented severe clinical worsening including asthenia, poor pain control, and reappearance of neurological symptoms with gait instability, dysphagia, left facial paralysis, spatial and temporal disorientation and short-term memory loss, summarizing a Performance Status (PS) of 3. Immediate CT brain scan showed no evidence of tumor progression, so, in a risky decision, we decided to optimize treatment including opioids and pregabalin, as well as corticosteroids at high doses (dexamethasone 4 mg twice a day during a week, followed by 4 mg per day during 1 week, and 2 mg per day during 1 week before stopping corticosteroids) in an attempt to control symptoms and improve PS until immunotherapy began. Afterwards, brain MRI confirmed our suspicions, detailing leptomeningeal enhancement adjacent to the left cerebellar hemisphere in the surgical area, as well as to the front area of the right cerebellar hemisphere, and also supratentorial involvement adjacent to left tentorium, with associated perilesional moderated edema (see Figure 2). These findings were confirmed by cerebrospinal fluid analysis.

**Figure 3:**
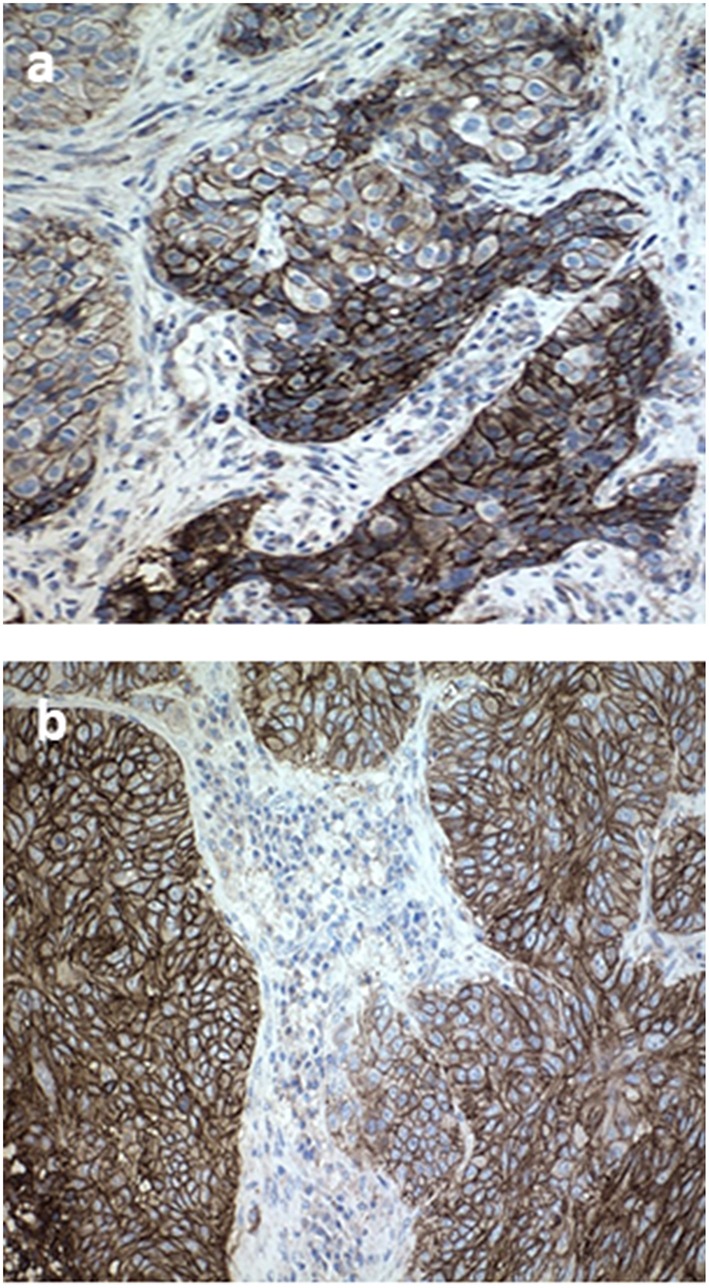
Tissue specimen of primary tumor sample and brain metastases. **(a)** Immunohistochemistry stained with PD-L1 primary antibody (28-8 pharmaDx; Dako) in a pretreated formalinfixed paraffin-embedded tissue of primary lung tumor before treatment, exhibiting strong membrane staining in 100% of tumor cells (20× magnification). **(b)** Cerebellar tissue specimen after complete resection.

Nivolumab was started on August, 24 at protocol dose of 3 mg/kg every 2 weeks. During first week, the patient was strictly controlled, objectifying progressive improvement of neurological symptoms, achieving assisted gait and progressively independent ambulation after two cycles, and full recovery of memory and level of consciousness after four. New brain MRI indicated complete leptomeningeal response after six nivolumab cycles, and CT body scan also confirmed lung complete response with no evidence of disease, both correlated with optimal PS1 and no evidence of neurological involvement except for residual left facial paralysis. In an attempt to hypothesize the possible reasons for complete response maintained over time, we requested the determination of PD-L1 level in the original tumor tissue. Surprisingly, high expression PD-L1 level was found in both lung and brain sample: tumor cells stained with PD-L1 primary antibody (28-8 pharmaDx; Dako) showed strong membrane staining in 100% of tumor cells (see Figure 3).

**Figure 4:**
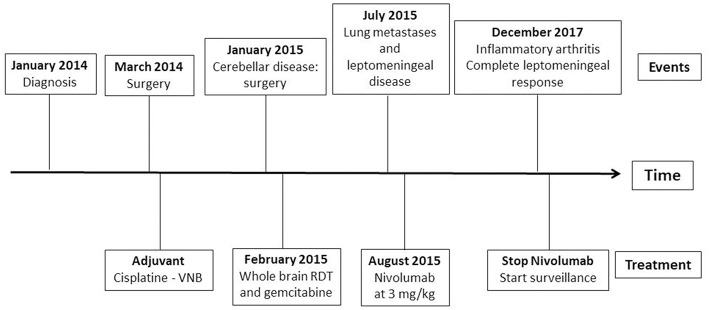
Timeline of events since the diagnosis and summary of administered treatments.

Nivolumab was well-tolerated during more than 2 years, with the only adverse effect being grade 1 diarrhea and grade 1 skin rash that did not require medical treatment for control. In December 2017, 2 years and 3 months after nivolumab was started, the patient complained of stiffness in the joints of hands, arms, ankles, and knees, added to inflammation and poorly controlled pain, suggesting inflammatory arthritis secondary to immunotherapy. Rheumatology started prednisone 10 mg twice a day with decreasing dosage, and we chose to stop treatment with nivolumab in the presence of a grade 3 adverse event and the need for treatment with high-dose glucocorticoids. After achieving a PFS of 28 months since introduction of immune blockage, nivolumab was not reinstated once acute arthritis had subsided. At the time of submission of this manuscript, the patient maintains excellent performance status with no neurological or pulmonary symptoms, either secondary adverse events related to nivolumab, and continues showing complete response in image tests including brain MRIs and CT body scans (see Figure 4).

## Discussion

Leptomeningeal carcinomatosis is an uncommon, but often aggressive, complication of solid tumors, specially melanoma, breast, and lung cancer, with an extremely poor prognosis. Venous or arterial haematogenous spread to choroid plexus, migration along perineural lymphatics or direct extension from adjacent pre-existing tumor lesions are the most accepted hypotheses for metastatic seeding of the leptomeninges. Data revealed a raising incidence of 3.8% in patients diagnosed of NSCLC, chiefly adenocarcinoma histology, with almost a third of them exhibiting concomitant brain metastases (11). This increase is probably due to an upgrowth in our therapeutic antitumor resources, leading median overall survival from 1–3 to 3–11 months, but also better neuro-imaging techniques and supportive cares. Systemic cytotoxic chemotherapy has small efficiency in this setting, probably related with inadequate penetration of the blood-brain barrier or inability to maintain optimal antitumor concentrations. Despite limited penetrance of gemcitabine and paclitaxel in central nervous system (CNS), both demonstrated consistent activity against brain metastases in a phase III trial, with an ORR of 28.9% and a median survival of 7.7 months (95% CI: 6.7–9.3), but no data about leptomeningeal disease were reported (12). Also pemetrexed, an accepted drug for non-squamous cell carcinoma, obtained valuable intracraneal response rate of 41.9% in spite of reduced CNS penetrance of less 5% (13).

In contrast, the results of the development of tailored drugs targeting oncogenic driver mutations in different pathways during the last decade suggest that tyrosine kinase inhibitors (TKIs) have significant activity in CNS, but data about leptomeningeal disease is scarce. Erlotinib and gefitinib, both Epidermal Growth Factor Receptor (EGFR) TKIs, were compared in a retrospective trial including 25 patients with leptomeningeal carcinomatosis, implying that erlotinib had superior capability of clear malignant cells from the cerebrospinal fluid (14). Moreover, pulsatile high-dose erlotinib obtained interesting clinical and radiological responses in 10 patients with leptomeningeal disease (15). Osimertinib, a third-generation EGFR-TKI, also demonstrated significant activity in CNS showing a PFS of 8.5 vs. 4.2 months in the chemotherapy arm with a hazard ratio of 0.32 (95% CI: 0.21–0.49) in a phase III trial (16). Recently, Flippot et al. (17) retrospectively analyzed 92 EGFR-mutated NSCLC patients which could use TKI rechallenge after progression, showing an OS since leptomeningeal disease of 6.1 and 7.6 months in case of TKI rechallenge. Moreover, almost 60% of patients showed clinical benefit with erlotinib after TKI first progression. In case of anaplastic lymphoma kinase (ALK) rearrangement, second generation inhibitors as Alectinib showed higher CNS response rate than standard of care crizotinib; in a case series of four patients with leptomeningeal disease progressing to crizotinib, three of them achieved clinical control and radiological reduction disease (18). Ceritinib, another second generation ALK inhibitor, have demonstrated optimal intracranial activity in patients progressing to crizotinib; phase II ASCEND-7 trial will further evaluate efficacy of ceritinib in brain and/or leptomeningeal metastases (19).

However, data about the role of immunotherapy in CNS is scarce, even more about leptomeningeal carcinomatosis. Because brain is a fragile organ with low recovery capability, immune response in CNS is highly regulated, protecting brain parenchyma from potentially aggressive immune reactions (20). Targeting inflammatory microenvironment of brain metastases, composed by microglia and T-cells, using immune-modulatory therapies represents a hard therapeutic challenge. Curiously, higher PDL-1 expression and decreased density of tumor infiltrating lymphocytes (TILs) are found in brain metastases regarding to primary tumor (20, 21). By protocol, patients harboring active brain metastases have not been included in pivotal trials of nivolumab, pembrolizumab, and atezolizumab, but patients with treated, stable brain metastases were allowed to be eligible. In Checkmate 017 (5), nine patients with CNS metastases were included in the nivolumab group and seven in the docetaxel group. This subgroup seemed to have obtained similar benefit in terms of OS but not including specific intracraneal image test. In Keynote 024 (7), the presence of brain metastases at baseline was 11.7% in the pembrolizumab group (6.6% in control group) and a trend toward benefit in both subgroups was observed. Moreover, pembrolizumab combined with chemotherapy also seemed to benefit patients with brain metastases at baseline; the combination was tested in 73 patients (17.8%) and achieved better results in the subgroup analysis with a Hazard Ratio of 0.36 (8). Finally, in the OAK study, 80 patients (10% of total population) with treated asymptomatic CNS metastases were included, demonstrating a hopeful OS of 20.1 months in patients treated with anti-PD-L1 atezolizumab against 11.9 months in the chemotherapy group (HR 0.54) (9).

It is unclear if immunotherapy is the leading responsible for brain metastases control disease, and the absence of prospective phase III trials evaluating this setting remains a remarkable impediment. Nonetheless, several papers have tried to determine the role of agents targeting PD-1 and PD-L1 in NSCLC with brain metastases, including leptomeningeal disease, but results are scarce. As an example, a recent analysis of 1,025 NSCLC patients (including 255 with brain metastases) treated with checkpoint inhibitors showed worse results in terms of PFS (1.7 vs. 2.1 months) and OS (8.6 vs. 11.4 months) for patients with intracraneal disease, but similar regarding to ORR (20.6 vs. 22.7%); leptomeningeal disease was an exclusion criteria (22). Gauvain et al. (23) analyzed in a retrospective study 43 patients with brain metastases, active CNS disease in 16 of them, treated with nivolumab. Primary endpoint was intracerebral objective response rate (9%), and extracranial RR was 11%; intracerebral disease-control rate was 51% (95% CI: 37–66%), appeared to be equivalent for extracerebral disease-control rate (47%), and achieved an intracraneal PFS of 3.9 months. Investigators concluded that nivolumab intracerebral activity is not inferior to extracerebral efficacy. Recently, Hendriks et al. (24) analyzed 19 NSCLC patients with leptomeningeal disease treated with Nivolumab or Pembrolizumab, achieving a 6-month PFS rate of 21% and 6-month OS rate of 36%, hypothesizing that good prognosis patients could benefit of immunotherapy in this setting. Dudnik et al. (25) presented five NSCLC patients with untreated intracranial disease, including two of them with leptomeningeal carcinomatosis, treated with nivolumab. They achieved one partial response and one stabilization, with a median time to intracraneal response of 7 weeks and a safety profile of immunotherapy according to previous data. Another clinical report of a patient with symptomatic leptomeningeal metastasis with auditory hallucinations showed clinical response, including disappearance of neurological symptoms, and partial response in MRI achieving a PFS of 7 months with second-line nivolumab, with no severe adverse events (26). According to Real World Data, the Italian Nivolumab Expanded Access Programme (27) included 409 NSCLC patients with asymptomatic and neurologically stable brain metastases, encompassing leptomeningeal carcinomatosis. Results showed a disease control rate (DRR) of 40% and ORR of 17%, with a median OS rate at 12 months of 35% for patients with CNS disease and 39% for all patients in the squamous NCLSC cohorts, suggesting reasonable activity of nivolumab in case of brain metastases and supporting the hypothesis of an overall benefit in the subgroup of CNS patients.

In our case, we have described a dramatic clinical response with nivolumab, getting neurological symptoms to subside in a few weeks, chiefly regarding a sustained complete radiological and clinical response during 3 years. The reason why our patient has an outstanding and express response to nivolumab remains unclear. We hypothesize that high PD-L1 expression level and TILs have a role as predictive biomarkers for immunotherapy, although their expression in brain metastases and especially in leptomeningeal disease is not studied yet (28). Berghoff et al. (29) investigated the role of TILs and their prognostic impact in patients with brain metastasis; they sustained that TIL infiltration was observed in 99% of brain metastasis samples, and the density of TIL correlated with an improved OS (15 vs. 6 months; *p* = 0.015 in CD3^+^; 15 vs. 11 months; *p* = 0.03 in CD8^+^). PD-L1 was represented in 28% of the samples, 26% of them were lung cancer brain metastasis, with no correlation with TIL density. Nevertheless, probably other agents as WBRT could play a role in this unexpected response to immunotherapy. Several reports of an abscopal effect of radiation therapy have been reported, suggesting a better extracranial control when radiation precedes immunotherapy. As an example, Ahmed et al. (30) obtained a 6 months rate of extracranial control disease of 57% when stereotactic radiation is given before or during immunotherapy, compared to 0% when immunotherapy preceded radiation. Further investigation is needed in this field.

Grade 3 toxicity of Nivolumab more than 2 years after first dose is a relevant episode. Immune-related adverse events are common and relatively well-tolerated, but they could even be a future predictor of response. Results in terms of PFS (9.2 vs. 4.8 months) and OS (Not Reach vs. 11.1 months) in a study of 134 NSCLC patients treated with Nivolumab were better in patients with immune related adverse events (31). Recently, Ricciuti et al. (32) retrospectively analyzed 195 NSCLC patients treated with Nivolumab, obtaining significant benefit in terms of PFS (5.7 vs. 2.0 months) and OS (17.8 vs. 4.0) in the subgroup which developed adverse events during treatment. Surprisingly, more than one adverse event was also a strong predictor of survival.

Immunotherapy outburst has led to a non-precedent overall survival in different primary tumors, so that responses to therapy are durable on time, represented by patients at the plateaus in the tail end of the Kaplan–Meier survival curves, which theoretically will benefit from longer responses. This percentage of patients who are considered “cured” could reach ~20–25% in several pivotal trials involving immune checkpoint inhibitors (33). As we mentioned above, in the 2-year outcomes from nivolumab pivotal trials in second-line NSCLC, OS rates were 23 and 29% with nivolumab vs. 8 and 16% with docetaxel, in, respectively, squamous and non-squamous (10). More remarkably, of previous confirmed responders in the nivolumab cohorts, 37% of patients with squamous NSCLC and 34% of patients with non-squamous NSCLC had ongoing responses after prolonged follow-up of at least 24 months, with no ongoing responses in the docetaxel group. Unfortunately, the way to identify these long-responders is currently unknown. The new immunotherapy predicting models managed the possibility of stopping the risk of progression disease at a certain point, rather than reach the 0 value in the group of survivals (34).

PD-L1 high expression of 100% is one of the most relevant facts. PD-L1 test is routinely used in terms of selecting first-line NSCLC treatment according to Keynote 024 (7). Nonetheless, this role is not clear in case of nivolumab. In the second-line nivolumab trial in squamous cell carcinoma (5), PD-L1 expression was neither prognostic nor predictive in terms of efficacy, and survival benefit of immunotherapy was observed in all subgroups, regardless of prespecified tumor PD-L1 expression levels (1, 5, and 10%). However, in non-squamous cell carcinoma (6), this benefit is clearly associated with PD-L1 expression, suggesting a predictive association between prespecified expression PD-L1 level and magnitude of clinical benefit, even though benefit of anti-PD-1 was observed in overall population. A recent updated survival data of 14 studies of immunotherapy in NSCLC revealed favorable data to PD-L1 positive patients (34). ORR in PD-L1 positive patients was 27.6%, contrasting with 12.1% of PD-L1 negative patients. Interestingly, the ORR increases with higher PD-L1 values, regardless of histology and first or second-line immunotherapy. Patients with PD-L1-positive tumors had also better OS (HR: 0.77; 95% CI: 0.67–0.89; *p* < 0.01). Absolut PD-L1 levels are not shown, but high overexpression of 100% is not reached. Probably future directions will be routed toward optimizing the selection of patients to PD-1/PD-L1 checkpoint blockade inhibitors. A recent study has studied the PD-L1 expression on circulating tumor cells (CTCs), which maintains characteristics of primary tumor and could serve to analyse therapeutic responses (35). CTC analysis could allow us to reveal dynamic changes in PD-L1, but also identify better candidates to benefit from immunotherapy. Investigators observed that there are different PD-L1 expression levels on CTCs, and PD-L1^high^ CTCs had a higher disease control rate (48%) than PD-L1^high^ CTC-negative patients (14%). Furthermore, PD-L1^high^ CTCs, both baseline and post-therapeutic quantity, could predict significance in terms of PFS.

To the best of our knowledge, this is the first report of complete and sustained leptomeningeal and pulmonary response to second line nivolumab in NSCLC, with complete response maintained over more than 3 years. Currently, this case comprehends a PFS of 43 months with no disease evidence in routine image tests, and future plans involve surveillance with clinical and image routine tests until progression, projecting a possible reintroduction of immunotherapy in this setting. We hope that our experience could contribute to learn more about the behavior of leptomeningeal carcinomatosis and expand the potential therapeutic spectrum of immunotherapy. Also, it supports the urgent necessity of more prospective trials helping us in the finding of a better definition of immunomarkers and identifying potential long-term responders.

## Ethics Statement

The treatment plan was approved by the Galician Local Research Ethics Committees (GGC-NIV-2018-01) and was executed in accordance with the Declaration of Helsinki, Good Clinical Practice, and local ethical and legal requirements. We obtained informed written consent from the patient authorizing publication of clinical case, which is attached to the medical records.

## Author Contributions

DA, PS, and JF contributed to the conception of the manuscript. MA, JG-M, and JF contributed to the patient care and management. IA contributed to analysis and histological interpretation of results. DA and CL drafted the paper. CR, DG-R, and JF supported final approval of the paper. All authors contributed to manuscript revision, read and approved the final version.

### Conflict of Interest Statement

The authors declare that the research was conducted in the absence of any commercial or financial relationships that could be construed as a potential conflict of interest.

## Abbreviations

AE: Adverse events
ALK: Anaplastic Lymphoma Kinasa
CNS: Central nervous system
CT: computed tomography
CTC: Circulating tumor cells
DRR: Disease control rate
EGFR: Epidermal growth factor receptor
HR: Hazard ratio
MRI: Magnetic resonance imaging
NSCLC: Non-small cell lung cancer
ORR: Overall response rate
OS: Overall survival
PD1: Programmed cell death-1
PD-L1: Programmed cell death ligand-1
PET: Positron emission tomography
PFS: Progression free survival
PS: Performance Status
TIL: Tumor infiltrating lymphocytes
TKI: tyrosine kinase inhibitors
VATS: Video-Assisted Thoracic Surgery.
